# Supplementary material for: A digital DNA system favours the superiority of unidirectional inheritance over ‘Lamarckian’ inheritance
Source: PLoS Comput Biol. 2025 Oct 7;21(10):e1012677. doi: 10.1371/journal.pcbi.1012677 (PMC12517530; doi:10.1371/journal.pcbi.1012677)
Supplement: S1 Fig — (PDF) [file pcbi.1012677.s001.pdf]

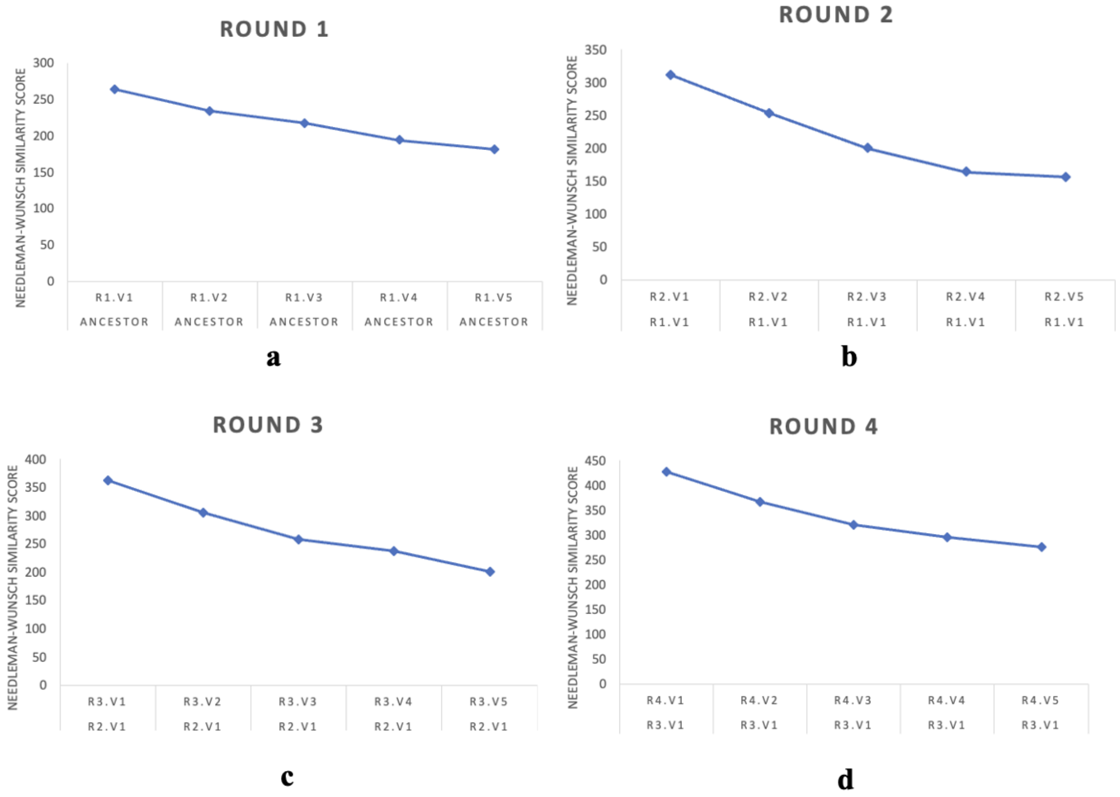

Figure S1. Music score comparison between the mutated audio files against the precursor audios using modified version of Needleman-Wunsch similarity calculation for the four-rounds. (**a**) When compared the music scores between the ancestor and five mutated generations in first round, the mutated version Round1.version1 (R1.V1) audio yielded the maximum similarity score of 264, and hence R1.V1 was chosen as the winning audio (**b**) Taking the winner of the previous round R1.V1 as the precursor, generated the next five mutated version in second round where R2.V2 yielded maximum similarity with R1.V1, with a similarity score of 312. Hence, R2.V2 was considered as the winner from the second round which then became the seed for the third round audio capture (**c**) Comparing the similarity between the mutated versions from third round against R2.V2, the maximum score obtained was 362, for R3.V1 (**d**) For the fourth round, R3.V1 was taken as the precursor and generated five mutated files with R4.V1 having maximum similarity score.
