# Supplementary material for: A digital DNA system favours the superiority of unidirectional inheritance over ‘Lamarckian’ inheritance
Source: PLoS Comput Biol. 2025 Oct 7;21(10):e1012677. doi: 10.1371/journal.pcbi.1012677 (PMC12517530; doi:10.1371/journal.pcbi.1012677)
Supplement: S1 Table — (DOCX) [file pcbi.1012677.s002.docx]

**Table S1.** MIDI standard

| **Octave** | **Note numbers** | | | | | | | | | | | |
| --- | --- | --- | --- | --- | --- | --- | --- | --- | --- | --- | --- | --- |
|  | C | C# | D | D# | E | F | F# | G | G# | A | A# | B |
| 0 | 0 | 1 | 2 | 3 | 4 | 5 | 6 | 7 | 8 | 9 | 10 | 11 |
| 1 | 12 | 13 | 14 | 15 | 16 | 17 | 18 | 19 | 20 | 21 | 22 | 23 |
| 2 | 24 | 25 | 26 | 27 | 28 | 29 | 30 | 31 | 32 | 33 | 34 | 35 |
| 3 | 36 | 37 | 38 | 39 | 40 | 41 | 42 | 43 | 44 | 45 | 46 | 47 |
| 4 | 48 | 49 | 50 | 51 | 52 | 53 | 54 | 55 | 56 | 57 | 58 | 59 |
| 5 | 60 | 61 | 62 | 63 | 64 | 65 | 66 | 67 | 68 | 69 | 70 | 71 |
| 6 | 72 | 73 | 74 | 75 | 76 | 77 | 78 | 79 | 80 | 81 | 82 | 83 |
| 7 | 84 | 85 | 86 | 87 | 88 | 89 | 90 | 91 | 92 | 93 | 94 | 95 |
| 8 | 96 | 97 | 98 | 99 | 100 | 101 | 102 | 103 | 104 | 105 | 106 | 107 |
| 9 | 108 | 109 | 110 | 111 | 112 | 113 | 114 | 115 | 116 | 117 | 118 | 119 |
| 10 | 120 | 121 | 122 | 123 | 124 | 125 | 126 | 127 |  |  |  |  |
