# Supplementary material for: A digital DNA system favours the superiority of unidirectional inheritance over ‘Lamarckian’ inheritance
Source: PLoS Comput Biol. 2025 Oct 7;21(10):e1012677. doi: 10.1371/journal.pcbi.1012677 (PMC12517530; doi:10.1371/journal.pcbi.1012677)
Supplement: S2 Table — (DOCX) [file pcbi.1012677.s003.docx]

**Table S2.** 4-letter DNA code for 16 notes x 4 durations

| **#** | **4-letter-codon** | **Note** | **Duration** |
| --- | --- | --- | --- |
| 1 | AAAA | 60 | 1 |
| 2 | AAAG | 62 | 1 |
| 3 | AAAC | 64 | 1 |
| 4 | AAAT | 65 | 1 |
| 5 | AAGA | 67 | 1 |
| 6 | AAGG | 69 | 1 |
| 7 | AAGC | 71 | 1 |
| 8 | AAGT | 72 | 1 |
| 9 | AACA | 74 | 1 |
| 10 | AACG | 76 | 1 |
| 11 | AACC | 77 | 1 |
| 12 | AACT | 79 | 1 |
| 13 | AATA | 81 | 1 |
| 14 | AATG | 83 | 1 |
| 15 | AATC | 84 | 1 |
| 16 | AATT | 86 | 1 |
| 17 | AGAA | 60 | 1 |
| 18 | AGAG | 62 | 1 |
| 19 | AGAC | 64 | 1 |
| 20 | AGAT | 65 | 1 |
| 21 | AGGA | 67 | 1 |
| 22 | AGGG | 69 | 1 |
| 23 | AGGC | 71 | 1 |
| 24 | AGGT | 72 | 1 |
| 25 | AGCA | 74 | 1 |
| 26 | AGCG | 76 | 1 |
| 27 | AGCC | 77 | 1 |
| 28 | AGCT | 79 | 1 |
| 29 | AGTA | 81 | 1 |
| 30 | AGTG | 83 | 1 |
| 31 | AGTC | 84 | 1 |
| 32 | AGTT | 86 | 1 |
| 33 | ACAA | 60 | 1 |
| 34 | ACAG | 62 | 1 |
| 35 | ACAC | 64 | 1 |
| 36 | ACAT | 65 | 1 |
| 37 | ACGA | 67 | 1 |
| 38 | ACGG | 69 | 1 |
| 39 | ACGC | 71 | 1 |
| 40 | ACGT | 72 | 1 |
| 41 | ACCA | 74 | 1 |
| 42 | ACCG | 76 | 1 |
| 43 | ACCC | 77 | 1 |
| 44 | ACCT | 79 | 1 |
| 45 | ACTA | 81 | 1 |
| 46 | ACTG | 83 | 1 |
| 47 | ACTC | 84 | 1 |
| 48 | ACTT | 86 | 1 |
| 49 | ATAA | 60 | 1 |
| 50 | ATAG | 62 | 1 |
| 51 | ATAC | 64 | 1 |
| 52 | ATAT | 65 | 1 |
| 53 | ATGA | 67 | 1 |
| 54 | ATGG | 69 | 1 |
| 55 | ATGC | 71 | 1 |
| 56 | ATGT | 72 | 1 |
| 57 | ATCA | 74 | 1 |
| 58 | ATCG | 76 | 1 |
| 59 | ATCC | 77 | 1 |
| 60 | ATCT | 79 | 1 |
| 61 | ATTA | 81 | 1 |
| 62 | ATTG | 83 | 1 |
| 63 | ATTC | 84 | 1 |
| 64 | ATTT | 86 | 1 |
| 65 | GAAA | 60 | 0.5 |
| 66 | GAAG | 62 | 0.5 |
| 67 | GAAC | 64 | 0.5 |
| 68 | GAAT | 65 | 0.5 |
| 69 | GAGA | 67 | 0.5 |
| 70 | GAGG | 69 | 0.5 |
| 71 | GAGC | 71 | 0.5 |
| 72 | GAGT | 72 | 0.5 |
| 73 | GACA | 74 | 0.5 |
| 74 | GACG | 76 | 0.5 |
| 75 | GACC | 77 | 0.5 |
| 76 | GACT | 79 | 0.5 |
| 77 | GATA | 81 | 0.5 |
| 78 | GATG | 83 | 0.5 |
| 79 | GATC | 84 | 0.5 |
| 80 | GATT | 86 | 0.5 |
| 81 | GGAA | 60 | 0.5 |
| 82 | GGAG | 62 | 0.5 |
| 83 | GGAC | 64 | 0.5 |
| 84 | GGAT | 65 | 0.5 |
| 85 | GGGA | 67 | 0.5 |
| 86 | GGGG | 69 | 0.5 |
| 87 | GGGC | 71 | 0.5 |
| 88 | GGGT | 72 | 0.5 |
| 89 | GGCA | 74 | 0.5 |
| 90 | GGCG | 76 | 0.5 |
| 91 | GGCC | 77 | 0.5 |
| 92 | GGCT | 79 | 0.5 |
| 93 | GGTA | 81 | 0.5 |
| 94 | GGTG | 83 | 0.5 |
| 95 | GGTC | 84 | 0.5 |
| 96 | GGTT | 86 | 0.5 |
| 97 | GCAA | 60 | 0.5 |
| 98 | GCAG | 62 | 0.5 |
| 99 | GCAC | 64 | 0.5 |
| 100 | GCAT | 65 | 0.5 |
| 101 | GCGA | 67 | 0.5 |
| 102 | GCGG | 69 | 0.5 |
| 103 | GCGC | 71 | 0.5 |
| 104 | GCGT | 72 | 0.5 |
| 105 | GCCA | 74 | 0.5 |
| 106 | GCCG | 76 | 0.5 |
| 107 | GCCC | 77 | 0.5 |
| 108 | GCCT | 79 | 0.5 |
| 109 | GCTA | 81 | 0.5 |
| 110 | GCTG | 83 | 0.5 |
| 111 | GCTC | 84 | 0.5 |
| 112 | GCTT | 86 | 0.5 |
| 113 | GTAA | 60 | 0.5 |
| 114 | GTAG | 62 | 0.5 |
| 115 | GTAC | 64 | 0.5 |
| 116 | GTAT | 65 | 0.5 |
| 117 | GTGA | 67 | 0.5 |
| 118 | GTGG | 69 | 0.5 |
| 119 | GTGC | 71 | 0.5 |
| 120 | GTGT | 72 | 0.5 |
| 121 | GTCA | 74 | 0.5 |
| 122 | GTCG | 76 | 0.5 |
| 123 | GTCC | 77 | 0.5 |
| 124 | GTCT | 79 | 0.5 |
| 125 | GTTA | 81 | 0.5 |
| 126 | GTTG | 83 | 0.5 |
| 127 | GTTC | 84 | 0.5 |
| 128 | GTTT | 86 | 0.5 |
| 129 | CAAA | 60 | 0.25 |
| 130 | CAAG | 62 | 0.25 |
| 131 | CAAC | 64 | 0.25 |
| 132 | CAAT | 65 | 0.25 |
| 133 | CAGA | 67 | 0.25 |
| 134 | CAGG | 69 | 0.25 |
| 135 | CAGC | 71 | 0.25 |
| 136 | CAGT | 72 | 0.25 |
| 137 | CACA | 74 | 0.25 |
| 138 | CACG | 76 | 0.25 |
| 139 | CACC | 77 | 0.25 |
| 140 | CACT | 79 | 0.25 |
| 141 | CATA | 81 | 0.25 |
| 142 | CATG | 83 | 0.25 |
| 143 | CATC | 84 | 0.25 |
| 144 | CATT | 86 | 0.25 |
| 145 | CGAA | 60 | 0.25 |
| 146 | CGAG | 62 | 0.25 |
| 147 | CGAC | 64 | 0.25 |
| 148 | CGAT | 65 | 0.25 |
| 149 | CGGA | 67 | 0.25 |
| 150 | CGGG | 69 | 0.25 |
| 151 | CGGC | 71 | 0.25 |
| 152 | CGGT | 72 | 0.25 |
| 153 | CGCA | 74 | 0.25 |
| 154 | CGCG | 76 | 0.25 |
| 155 | CGCC | 77 | 0.25 |
| 156 | CGCT | 79 | 0.25 |
| 157 | CGTA | 81 | 0.25 |
| 158 | CGTG | 83 | 0.25 |
| 159 | CGTC | 84 | 0.25 |
| 160 | CGTT | 86 | 0.25 |
| 161 | CCAA | 60 | 0.25 |
| 162 | CCAG | 62 | 0.25 |
| 163 | CCAC | 64 | 0.25 |
| 164 | CCAT | 65 | 0.25 |
| 165 | CCGA | 67 | 0.25 |
| 166 | CCGG | 69 | 0.25 |
| 167 | CCGC | 71 | 0.25 |
| 168 | CCGT | 72 | 0.25 |
| 169 | CCCA | 74 | 0.25 |
| 170 | CCCG | 76 | 0.25 |
| 171 | CCCC | 77 | 0.25 |
| 172 | CCCT | 79 | 0.25 |
| 173 | CCTA | 81 | 0.25 |
| 174 | CCTG | 83 | 0.25 |
| 175 | CCTC | 84 | 0.25 |
| 176 | CCTT | 86 | 0.25 |
| 177 | CTAA | 60 | 0.25 |
| 178 | CTAG | 62 | 0.25 |
| 179 | CTAC | 64 | 0.25 |
| 180 | CTAT | 65 | 0.25 |
| 181 | CTGA | 67 | 0.25 |
| 182 | CTGG | 69 | 0.25 |
| 183 | CTGC | 71 | 0.25 |
| 184 | CTGT | 72 | 0.25 |
| 185 | CTCA | 74 | 0.25 |
| 186 | CTCG | 76 | 0.25 |
| 187 | CTCC | 77 | 0.25 |
| 188 | CTCT | 79 | 0.25 |
| 189 | CTTA | 81 | 0.25 |
| 190 | CTTG | 83 | 0.25 |
| 191 | CTTC | 84 | 0.25 |
| 192 | CTTT | 86 | 0.25 |
| 193 | TAAA | 60 | 0.125 |
| 194 | TAAG | 62 | 0.125 |
| 195 | TAAC | 64 | 0.125 |
| 196 | TAAT | 65 | 0.125 |
| 197 | TAGA | 67 | 0.125 |
| 198 | TAGG | 69 | 0.125 |
| 199 | TAGC | 71 | 0.125 |
| 200 | TAGT | 72 | 0.125 |
| 201 | TACA | 74 | 0.125 |
| 202 | TACG | 76 | 0.125 |
| 203 | TACC | 77 | 0.125 |
| 204 | TACT | 79 | 0.125 |
| 205 | TATA | 81 | 0.125 |
| 206 | TATG | 83 | 0.125 |
| 207 | TATC | 84 | 0.125 |
| 208 | TATT | 86 | 0.125 |
| 209 | TGAA | 60 | 0.125 |
| 210 | TGAG | 62 | 0.125 |
| 211 | TGAC | 64 | 0.125 |
| 212 | TGAT | 65 | 0.125 |
| 213 | TGGA | 67 | 0.125 |
| 214 | TGGG | 69 | 0.125 |
| 215 | TGGC | 71 | 0.125 |
| 216 | TGGT | 72 | 0.125 |
| 217 | TGCA | 74 | 0.125 |
| 218 | TGCG | 76 | 0.125 |
| 219 | TGCC | 77 | 0.125 |
| 220 | TGCT | 79 | 0.125 |
| 221 | TGTA | 81 | 0.125 |
| 222 | TGTG | 83 | 0.125 |
| 223 | TGTC | 84 | 0.125 |
| 224 | TGTT | 86 | 0.125 |
| 225 | TCAA | 60 | 0.125 |
| 226 | TCAG | 62 | 0.125 |
| 227 | TCAC | 64 | 0.125 |
| 228 | TCAT | 65 | 0.125 |
| 229 | TCGA | 67 | 0.125 |
| 230 | TCGG | 69 | 0.125 |
| 231 | TCGC | 71 | 0.125 |
| 232 | TCGT | 72 | 0.125 |
| 233 | TCCA | 74 | 0.125 |
| 234 | TCCG | 76 | 0.125 |
| 235 | TCCC | 77 | 0.125 |
| 236 | TCCT | 79 | 0.125 |
| 237 | TCTA | 81 | 0.125 |
| 238 | TCTG | 83 | 0.125 |
| 239 | TCTC | 84 | 0.125 |
| 240 | TCTT | 86 | 0.125 |
| 241 | TTAA | 60 | 0.125 |
| 242 | TTAG | 62 | 0.125 |
| 243 | TTAC | 64 | 0.125 |
| 244 | TTAT | 65 | 0.125 |
| 245 | TTGA | 67 | 0.125 |
| 246 | TTGG | 69 | 0.125 |
| 247 | TTGC | 71 | 0.125 |
| 248 | TTGT | 72 | 0.125 |
| 249 | TTCA | 74 | 0.125 |
| 250 | TTCG | 76 | 0.125 |
| 251 | TTCC | 77 | 0.125 |
| 252 | TTCT | 79 | 0.125 |
| 253 | TTTA | 81 | 0.125 |
| 254 | TTTG | 83 | 0.125 |
| 255 | TTTC | 84 | 0.125 |
| 256 | TTTT | 86 | 0.125 |
