# Supplementary material for: A digital DNA system favours the superiority of unidirectional inheritance over ‘Lamarckian’ inheritance
Source: PLoS Comput Biol. 2025 Oct 7;21(10):e1012677. doi: 10.1371/journal.pcbi.1012677 (PMC12517530; doi:10.1371/journal.pcbi.1012677)
Supplement: S3 Table — (DOCX) [file pcbi.1012677.s004.docx]

**Table S3.** Bitscore calculations for purifying selection.

| **Experiment-round *(n)*** | **Bitscore*(n-1)-***  *Round(n-1) vs. Round(n-1)* | **Bitscore*(n)*-**  *Round(n-1) vs. Round(n)* | **𝛥 Bitscore**  Bitscore*(n-1)* - Bitscore*(n)* | **Length** |
| --- | --- | --- | --- | --- |
| Round 1 | 1240  *(Ancestor vs. Ancestor)* | 741  *(Ancestor vs. Round1-winner)* | 499 | 304 |
| Round 2 | 1520  *(Round1-winner vs. Round1-winner)* | 779  *(Round1-winner vs. Round2-winner)* | 741 | 361 |
| Round 3 | 1700  *(Round2-winner vs. Round2-winner)* | 959  *(Round2-winner vs. Round3-winner)* | 741 | 400 |
| Round 4 | 1960  *(Round3-winner vs. Round3-winner)* | 1119  *(Round3-winner vs. Round4-winner)* | 841 | 419 |
