# Supplementary material for: A digital DNA system favours the superiority of unidirectional inheritance over ‘Lamarckian’ inheritance
Source: PLoS Comput Biol. 2025 Oct 7;21(10):e1012677. doi: 10.1371/journal.pcbi.1012677 (PMC12517530; doi:10.1371/journal.pcbi.1012677)
Supplement: S3 File — (PDF) [file pcbi.1012677.s008.pdf]

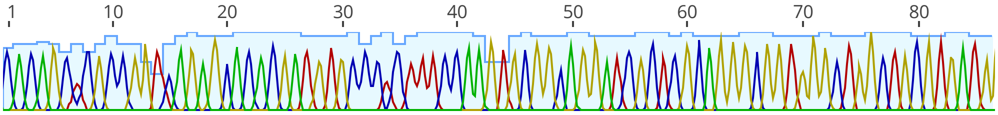

FWD 4_22G777A-1_M13R.ab1 CTCTGCACGCCGGACTGTGCTCTCGTAGAGCCCACAAACCTTGAGCGGCTGGTACGCACGCTGGGCGGAGGGCAGGCAGCGAGTAG

REV round1-version1_poll_data ACAAACCTTGAGCGGCTGGTACGCACGCTGGGCGGAGGGCAGGCAGCGAGTAG

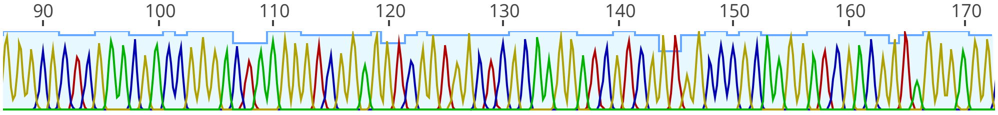

FWD 4_22G777A-1_M13R.ab1 GGGCGCACGTTCGTCCGGGTCATTGGGACGGTGGACGGAGGCACGCTTGGTACGACGGAGGCCCGCTTGGTACGTCGGATGGGTGG

REV round1-version1_poll_data GGGCGCACGTTCGTCCGGGTCATTGGGACGGTGGACGGAGGCACGCTTGGTACGACGGAGGCCCGCTTGGTACGTCGGATGGGTGG

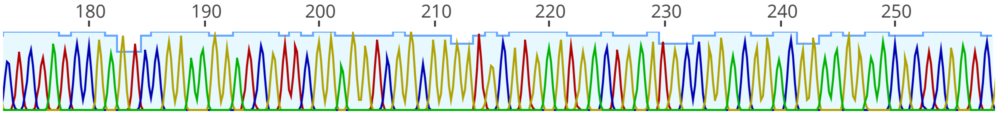

FWD 4_22G777A-1_M13R.ab1 CACATACCGTGACCGGTTGGTACGAACGGTGGACGGCGGGGAGCGAGTGAGTCAGTGAGCCGGTCGGTCGGTTGGTTCGCACGTAC

REV round1-version1_poll_data CACATACCGTGACCGGTTGGTACGAACGGTGGACGGCGGGGAGCGAGTGAGTCAGTGAGCCGGTCGGTCGGTTGGTTCGCACGTAC

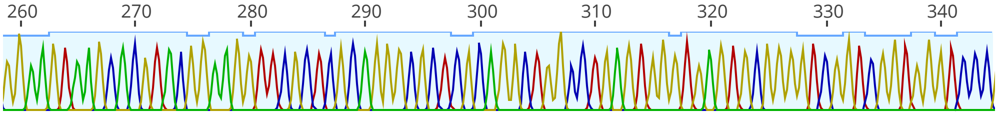

FWD 4_22G777A-1_M13R.ab1 GGTTGATTGCTCGATCGGTTGGAACGCACGCTGGGCGCACGCTGGCAGGCCAGTGAGGGAGTGAGCGGGGACGGACGGAGGGACCC

REV round1-version1_poll_data GGTTGATTGCTCGATCGGTTGGAACGCACGCTGGGCGCACGCTGGCAGGCCAGTGAGGGAGTGAGCGGGGACGGACGGA

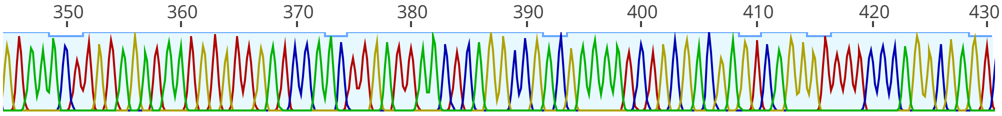

FWD 4_22G777A-1_M13R.ab1 GATTTCAAGATGTATTAGAGAAGTACCTTCAAGTAATTCACTGGCCGTCGTTTTACAGCGTCGTGACTGGGAAAACCCTGGCGTGA

REV round1-version1_poll_data

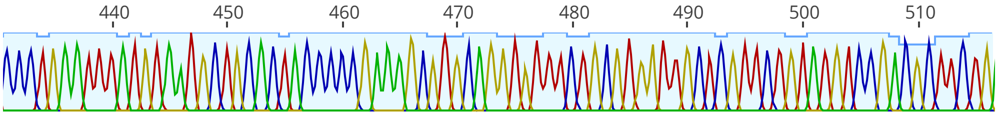

FWD 4_22G777A-1_M13R.ab1 CCCAGTTAAATAGATTAGCAGCTCATCCCCCGTTTGCGAGCTGGAGAAACAGCGAGGAAGCACGAACAGATAGACCGTCGCAACAG

REV round1-version1_poll_data

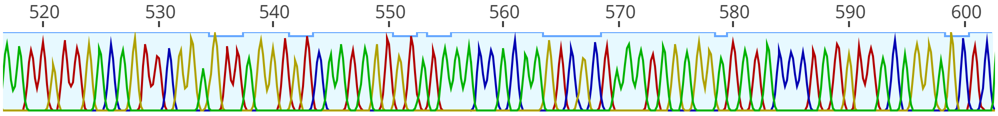

FWD 4_22G777A-1_M13R.ab1 TTAAGAAGTCTGAACGGTGAATGGAGACTTATGAGATATTTCCTCTTGACGCATTTATGTGGTATATCCCATAGAATCTGGTGCAC

REV round1-version1_poll_data

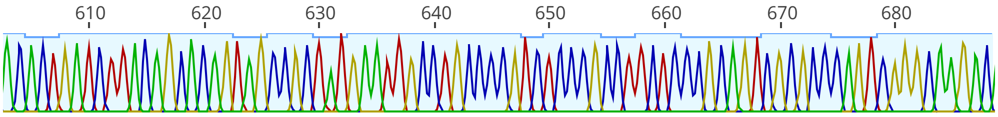

FWD 4_22G777A-1_M13R.ab1 TCTCAGTACAATCTGCTCTGATGCCGCATAGTTAAGCCAGCCCCGACACCCGCCAACACCCGCTGACGCGCCCTGACGGGCTTGTC

REV round1-version1_poll_data

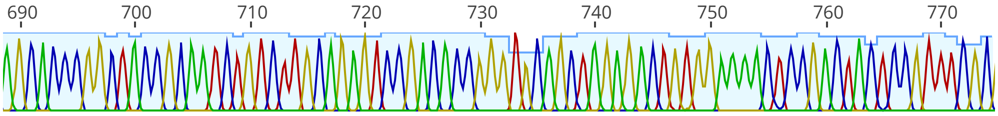

FWD 4_22G777A-1_M13R.ab1 TGCTCCCGGCATCCGCTTACAGACAAGCTGTGACCGTCTCCGGGAGCTGCATGTGTCAGAGGTTTTCACCGTCATCACCGAAACGC

REV round1-version1_poll_data

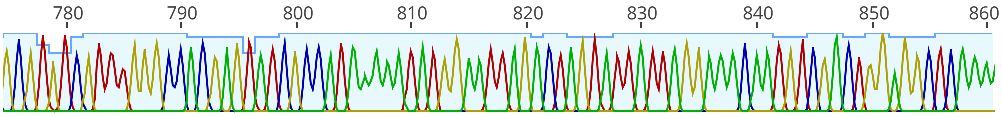

FWD 4_22G777A-1_M13R.ab1 GCGAGACGAAAGGGCCTCGTGATACGCCTATTTTTATAGGTTAATGTCATGATAATAATGGTTTCTTAGACGTCAGGTGGCACTTT

REV round1-version1_poll_data

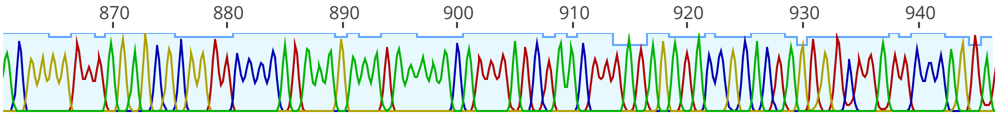

FWD 4_22G777A-1_M13R.ab1 TCGGGGAAATGTGCGCGGAACCCCTATTTGTTTATTTTTCTAAATACATTCAAATATGTATCCGCTCATGAGACAATAACCCTGAT

REV round1-version1_poll_data

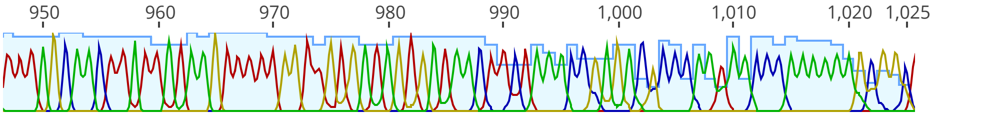

FWD 4_22G777A-1_M13R.ab1 AAATGCTTCAATAATATTGAAAAAGGAAGAGTATGAGTATTCAACATTTCCGTGTCGCCCTTATTCCCTTTTTTGCGGC

REV round1-version1_poll_data

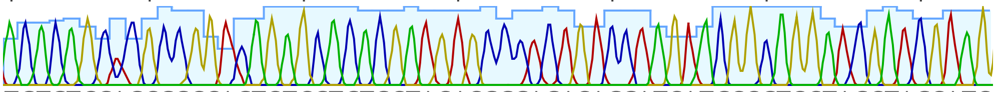

FWD HC00558165_2 TCTCTGCACGCCGGACTGTGCTCTCGTAGAGCCCACAGACCATGATCGGCTGGTACGTACGATG
REV round2-version1_poll_data ACAGACCATGATCGGCTGGTACGTACGATG

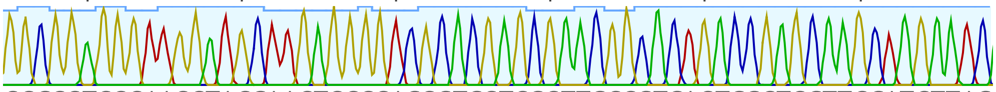

FWD HC00558165_2 GGCGGTGGGAAGGTAGCAAGTGGGGACGCTCGTGCGTTCGGCTCAGTCCGTGCTTGCATGTTAC
REV round2-version1_poll_data GGCGGTGGGAAGGTAGCAAGTGGGGACGCTCGTGCGTTCGGCTCAGTCCGTGCTTGCATGTTAC

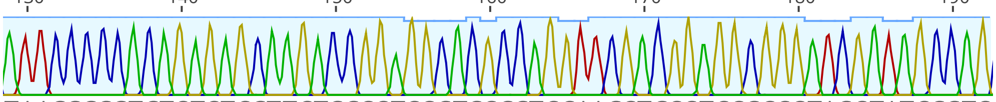

FWD HC00558165_2 TAACCCCCTCTGTGTGCTTGTCCGGTGGCTCGCCTGGAACGTCGGTGGCGGGTACGTATGCCTG
REV round2-version1_poll_data TAACCCCCTCTGTGTGCTTGTCCGGTGGCTCGCCTGGAACGTCGGTGGCGGGTACGTATGCCTG

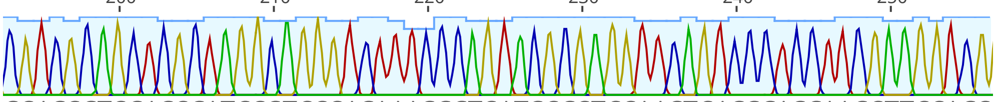

FWD HC00558165_2 CGACGCTGCACGCATGGCTGGGACAAACCCTGATCGCGTGGAACTGACCCACCAACGTTGGACG
REV round2-version1_poll_data CGACGCTGCACGCATGGCTGGGACAAACCCTGATCGCGTGGAACTGACCCACCAACGTTGGACG

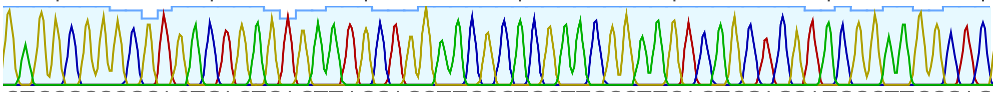

FWD HC00558165_2 GTGGCGGGCGAGTCAGTGAGTTAGCAGGTTCGCTCGTTCGGTTGACTGCACGATCGGTTGGCAC
REV round2-version1_poll_data GTGGCGGGCGAGTCAGTGAGTTAGCAGGTTCGCTCGTTCGGTTGACTGCACGATCGGTTGGCAC

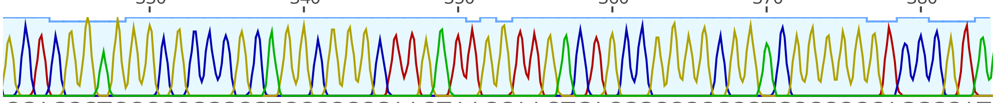

FWD HC00558165_2 GCACGGTGGGCGCCCGCTGGCGGGCAAGTAAGGAAGTCAGCCGGGGCGGTCGGGGGGACCCGAT
REV round2-version1_poll_data GCACGGTGGGCGCCCGCTGGCGGGCAAGTAAGGAAGTCAGCCGGGGCGGTCGGG

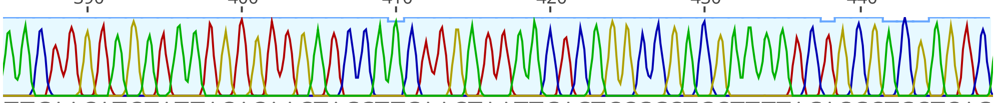

FWD HC00558165_2 TTCAAGATGTATTAGAGAAGTACCTTCAAGTAATTCACTGGCCGTCGTTTTACAGCGTCGTGAC
REV round2-version1_poll_data

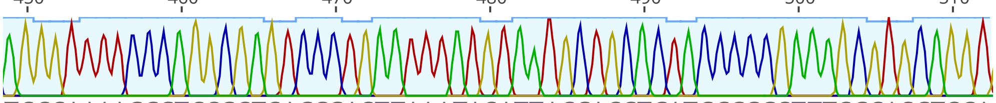

FWD HC00558165_2 TGGGAAAACCCTGGCGTGACCCAGTTAAATAGATTAGCAGCTCATCCCCCGTTTGCGAGCTGGA
REV round2-version1_poll_data

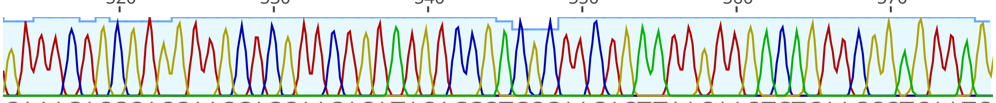

FWD HC00558165_2 GAAACAGCGAGGAAGCACGAACAGATAGACCGTCGCAACAGTTAAGAAGTCTGAACGGTGAATG
REV round2-version1_poll_data

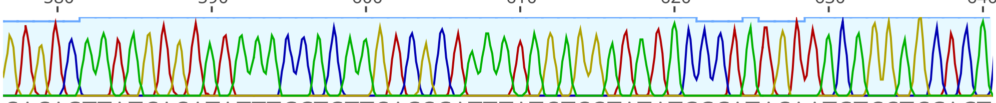

FWD HC00558165_2 GAGACTTATGAGATATTTCCTCTTGACGCATTTATGTGGTATATCCCATAGAATCTGGTGCACT
REV round2-version1_poll_data

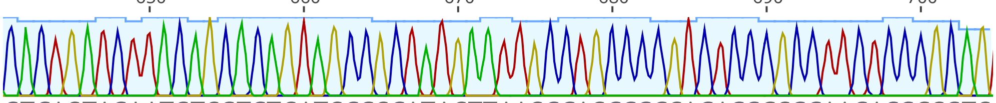

FWD HC00558165_2 CTCAGTACAATCTGCTCTGATGCCGCATAGTTAAGCCAGCCCCGACACCCGCCAACACCCGCTG
REV round2-version1_poll_data

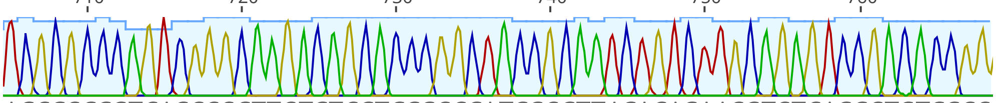

FWD HC00558165_2 ACGCGCCCTGACGGGCTTGTCTGCTCCCGGCATCCGCTTACAGACAAGCTGTGACCGTCTCCGG
REV round2-version1_poll_data

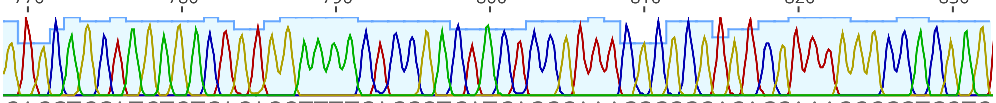

FWD HC00558165_2 GAGCTGCATGTGTCAGAGGTTTTCACCGTCATCACCGAAACGCGCGAGACGAAAGGGCCTCGTG
REV round2-version1_poll_data

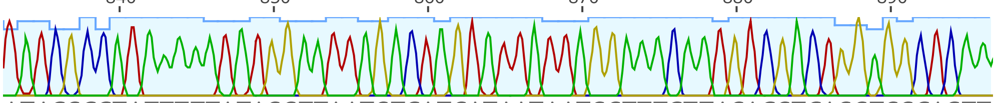

FWD HC00558165_2 ATACGCCTATTTTTATAGGTTAATGTCATGATAATAATGGTTTCTTAGACGTCAGGTGGCACTT
REV round2-version1_poll_data

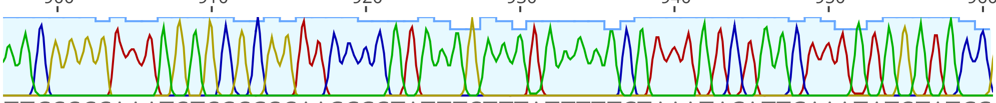

FWD HC00558165_2 TTCGGGGAAATGTGCGCGGAACCCCTATTTGTTTATTTTTCTAAATACATTCAAATATGTATCC
REV round2-version1_poll_data

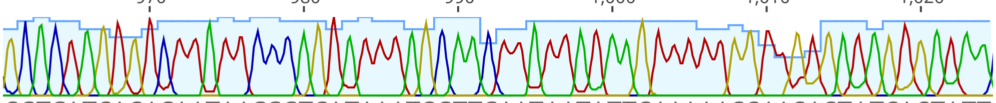

FWD HC00558165_2 GCTCATGAGACAATAACCCTGATAAATGCTTCAATAATATTGAAAAAGGAAGAGTATGAGTATT
REV round2-version1_poll_data

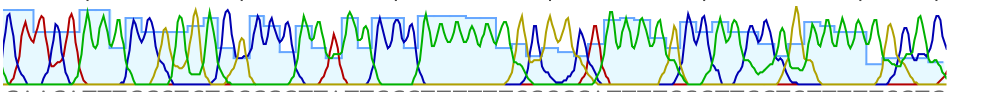

FWD HC00558165_2 CAACATTTCCGTGTCGCCCTTATTCCCTTTTTTGCGGCATTTTGCCTTCCTGTTTTTGCTC
REV round2-version1_poll_data

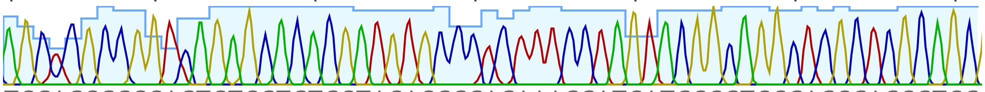

FWD HC00558165_3 TGCACGCCGGACTGTGCTCTCGTAGAGCCCACAAACCATGATCGGCTGGCACGCACGCTGC
REV round3-version1_poll_data ACAAACCATGATCGGCTGGCACGCACGCTGC

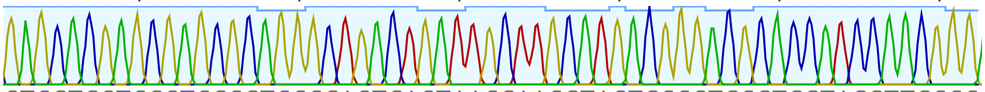

FWD HC00558165_3 GTGCTCGTGCGTGCGCTGGGCAGTCAGTAAGCAAGCTAGTCGGGTCGCTCCTACCTTCGGG
REV round3-version1_poll_data GTGCTCGTGCGTGCGCTGGGCAGTCAGTAAGCAAGCTAGTCGGGTCGCTCCTACCTTCGGG

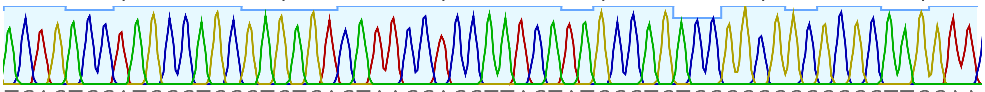

FWD HC00558165_3 TCAGTCCATGCCTGCGTGTGACTAACCACCTTACTATGCCTGTCCGGCGGCGCGCTTGGAA
REV round3-version1_poll_data TCAGTCCATGCCTGCGTGTGACTAACCACCTTACTATGCCTGTCCGGCGGCGCGCTTGGAA

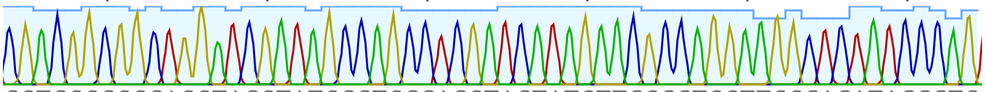

FWD HC00558165_3 CGTCGGCGGCAGGTACGTATGCCTGCCACGTACTATGTTCGCCTGGTTGGCACATACCCTG
REV round3-version1_poll_data CGTCGGCGGCAGGTACGTATGCCTGCCACGTACTATGTTCGCCTGGTTGGCACATACCCTG

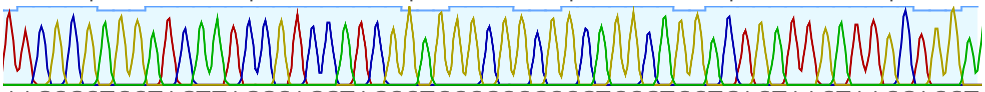

FWD HC00558165_3 AACGCGTGGTACTTACCGACCTACGGTGGGCGGCGGCTGGCTGGTCAGTAAGTAAGCAGGT
REV round3-version1_poll_data AACGCGTGGTACTTACCGACCTACGGTGGGCGGCGGCTGGCTGGTCAGTAAGTAAGCAGGT

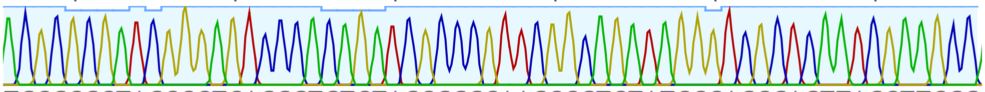

FWD HC00558165_3 TCGCGCGTACGGGTGACCCTCTGTACGCCCGAACGGCTGTATGGGACGCACTTACGTTGCC
REV round3-version1_poll_data TCGCGCGTACGGGTGACCCTCTGTACGCCCGAACGGCTGTATGGGACGCACTTACGTTGCC

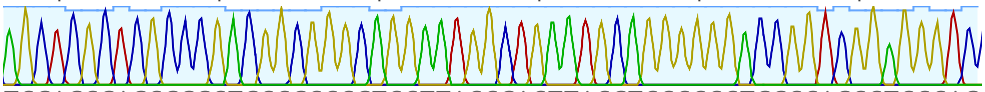

FWD HC00558165_3 TGCACGCACGCCCGTCGGCGGGCTGGTTAGGCAGTTAGCTGGGGGGTCCGGACGGTGGGAC
REV round3-version1_poll_data TGCACGCACGCCCGTCGGCGGGCTGGTTAGGCAGTTAGCTGGGGGGTCCGGACGGT

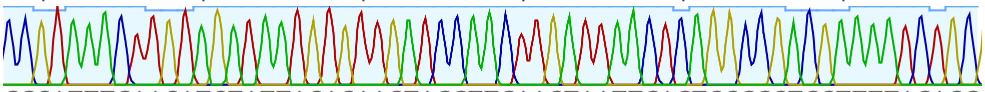

FWD HC00558165_3 CCGATTTCAAGATGTATTAGAGAAGTACCTTCAAGTAATTCACTGGCCGTCGTTTTACAGC
REV round3-version1_poll_data

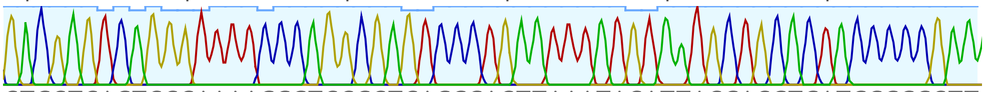

FWD HC00558165_3 GTCGTGACTGGGAAAACCCTGGCGTGACCCAGTTAAATAGATTAGCAGCTCATCCCCCGTT
REV round3-version1_poll_data

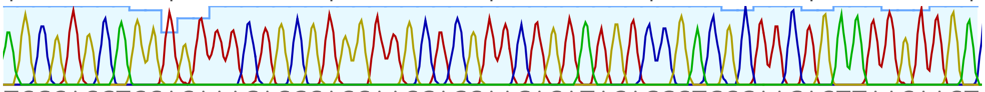

FWD HC00558165_3 TGCGAGCTGGAGAAACAGCGAGGAAGCACGAACAGATAGACCGTCGCAACAGTTAAGAAGT
REV round3-version1_poll_data

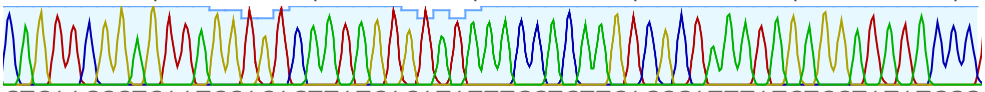

FWD HC00558165_3 CTGAACGGTGAATGGAGACTTATGAGATATTTCCTCTTGACGCATTTATGTGGTATATCCC
REV round3-version1_poll_data

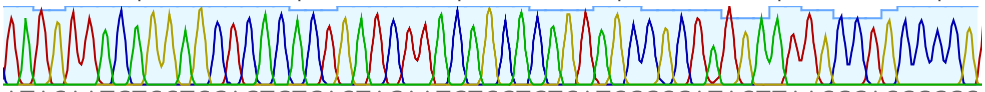

FWD HC00558165_3 ATAGAATCTGGTGCACTCTCAGTACAATCTGCTCTGATGCCGCATAGTTAAGCCAGCCCCG
REV round3-version1_poll_data

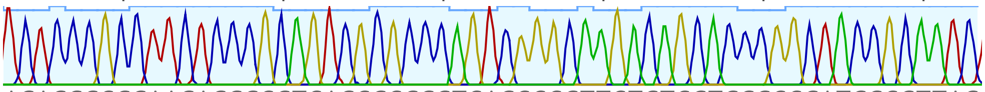

FWD HC00558165_3 ACACCCGCCAACACCCGCTGACGCGCCCTGACGGGCTTGTCTGCTCCCGGCATCCGCTTAC
REV round3-version1_poll_data

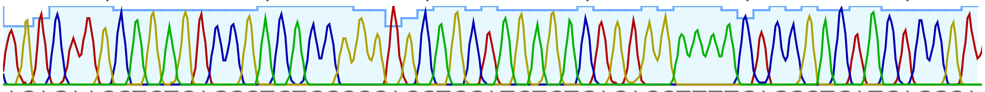

FWD HC00558165_3 AGACAAGCTGTGACCGTCTCCGGGAGCTGCATGTGTCAGAGGTTTTCACCGTCATCACCGA
REV round3-version1_poll_data

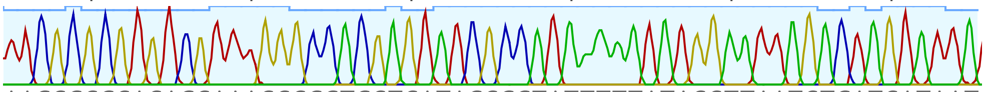

FWD HC00558165_3 AACGCGCGAGACGAAAGGGCCTCGTGATACGCCTATTTTTATAGGTTAATGTCATGATAAT
REV round3-version1_poll_data

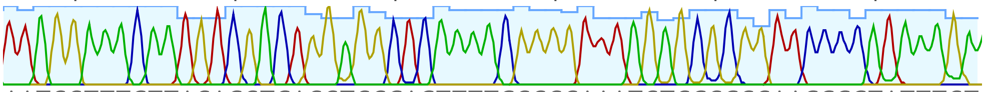

FWD HC00558165_3 AATGGTTTCTTAGACGTCAGGTGGCACTTTTCGGGGAAATGTGCGCGGAACCCCTATTTGT
REV round3-version1_poll_data

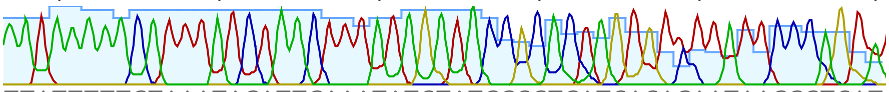

FWD HC00558165_3 TTATTTTTCTAAATACATTCAAATATGTATCCGCTCATGAGACAATAACCCTGAT
REV round3-version1_poll_data

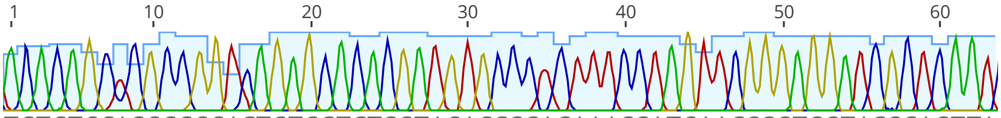

FWD HC00558165_4 TCTCTGCACGCCGGACTGTGCTCTCGTAGAGCCCACAAACCATGAACGGGTGGTACGCACTTA

REV round4-version1_poll_data ACAAACCATGAACGGGTGGTACGCACTTA

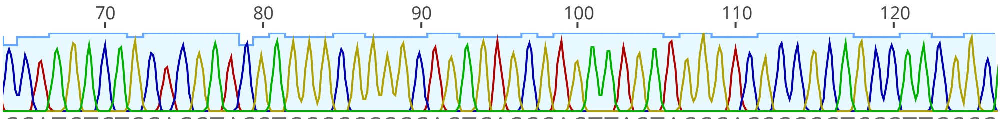

FWD HC00558165_4 CCATGTCTGCACGTACGTGGGCGGGGCAGTGAGCGAGTTAGTAGGGACGCCGCTGCCTTCGGC

REV round4-version1_poll_data CCATGTCTGCACGTACGTGGGCGGGGCAGTGAGCGAGTTAGTAGGGACGCCGCTGCCTTCGGC

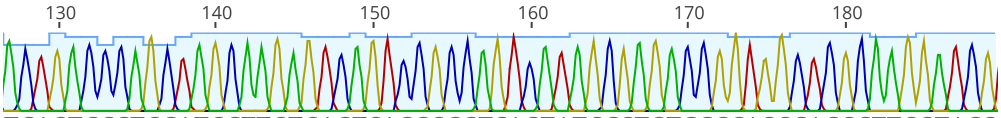

FWD HC00558165_4 TCAGTCCCTGCATGCTTGTGACTGACCGCCTGACTATGCGTGTCCGGAGGCACGCTTGGTACG

REV round4-version1_poll_data TCAGTCCCTGCATGCTTGTGACTGACCGCCTGACTATGCGTGTCCGGAGGCACGCTTGGTACG

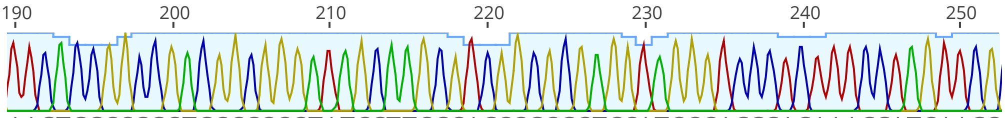

FWD HC00558165_4 AACTCCGGCCGTCGGCGGGTATGCTTGCGACGGCGCGTGGATGGGACCCACAAACCATGAACG

REV round4-version1_poll_data AACTCCGGCCGTCGGCGGGTATGCTTGCGACGGCGCGTGGATGGGACCCACAAACCATGAACG

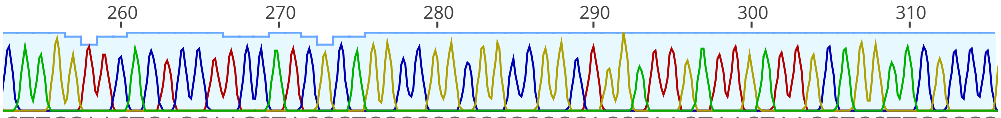

FWD HC00558165_4 CTTGGAACTCACCAACCTACGCTGGCCGGCGGCCGGCAGGTAAGTAAGTAAGCTGGTTCGCCC

REV round4-version1_poll_data CTTGGAACTCACCAACCTACGCTGGCCGGCGGCCGGCAGGTAAGTAAGTAAGCTGGTTCGCCC

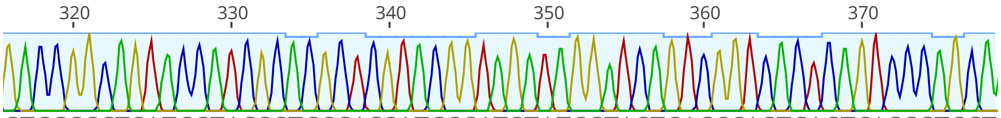

FWD HC00558165_4 GTCCGGCTGATCCTACGCTCGCACGATCGGATGTATGGTACTGACGGACTCACTGACCCTGCT

REV round4-version1_poll_data GTCCGGCTGATCCTACGCTCGCACGATCGGATGTATGGTACTGACGGACTCACTGACCCTGCT

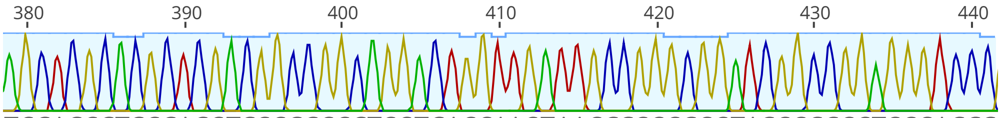

FWD HC00558165_4 TGCACGCTCGCACGTCGGCCGGCTGGTCAGGAAGTAAGCCGGGCGGTACGGCCGGTGGGACCC

REV round4-version1_poll_data TGCACGCTCGCACGTCGGCCGGCTGGTCAGGAAGTAAGCCGGGCGGTACGGCCGGT

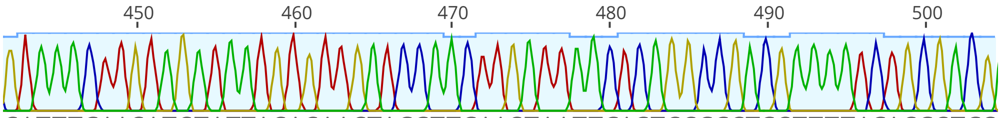

FWD HC00558165_4 GATTTCAAGATGTATTAGAGAAGTACCTTCAAGTAATTCACTGGCCGTCGTTTTACAGCGTCG

REV round4-version1_poll_data

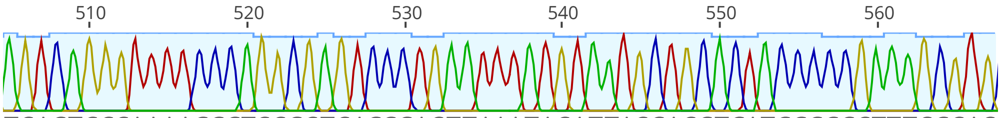

FWD HC00558165_4 TGACTGGGAAAACCCTGGCGTGACCCAGTTAAATAGATTAGCAGCTCATCCCCCGTTTGCGAG

REV round4-version1_poll_data

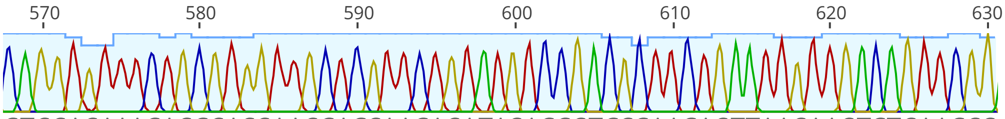

FWD HC00558165_4 CTGGAGAAACAGCGAGGAAGCACGAACAGATAGACCGTCGCAACAGTTAAGAAGTCTGAACGG

REV round4-version1_poll_data

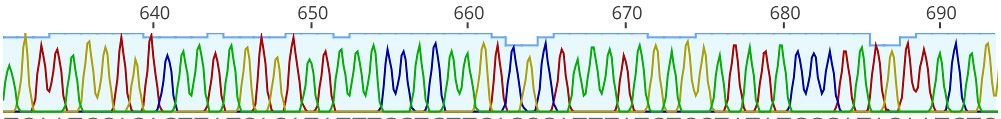

FWD HC00558165_4 TGAATGGAGACTTATGAGATATTTCCTCTTGACGCATTTATGTGGTATATCCCATAGAATCTG

REV round4-version1_poll_data

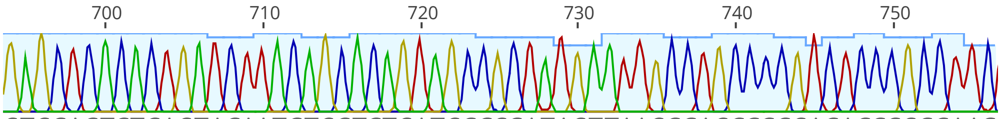

FWD HC00558165_4 GTGCACTCTCAGTACAATCTGCTCTGATGCCGCATAGTTAAGCCAGCCCCGACACCCGCCAAC

REV round4-version1_poll_data

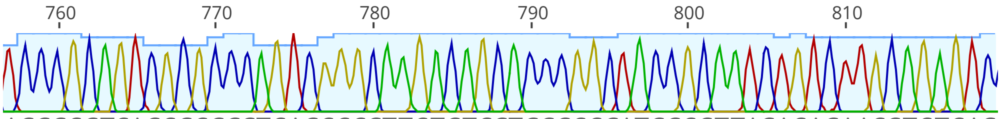

FWD HC00558165_4 ACCCGCTGACGCGCCCTGACGGGCTTGTCTGCTCCCGGCATCCGCTTACAGACAAGCTGTGAC

REV round4-version1_poll_data

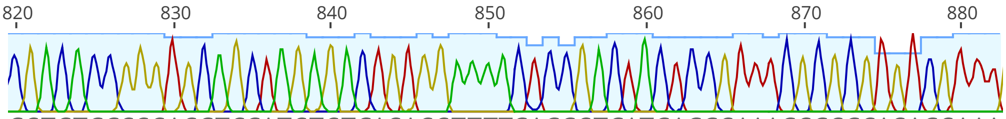

FWD HC00558165_4 CGTCTCCGGGAGCTGCATGTGTCAGAGGTTTTCACCGTCATCACCGAAACGCGCGAGACGAAA

REV round4-version1_poll_data

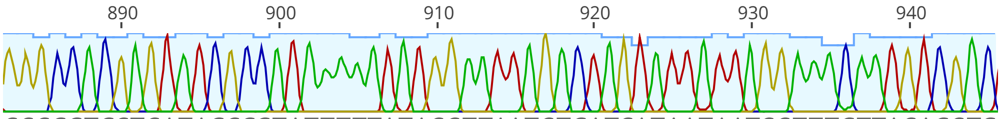

FWD HC00558165_4 GGGCCTCGTGATACGCCTATTTTTATAGGTTAATGTCATGATAATAATGGTTTCTTAGACGTC

REV round4-version1_poll_data

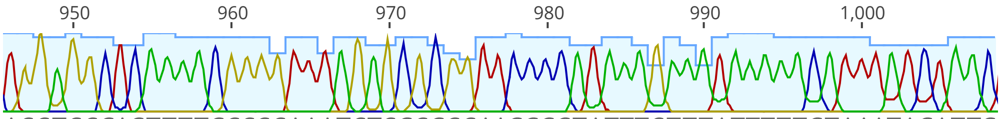

FWD HC00558165_4 AGGTGGCACTTTTCGGGGAAATGTGCGCGGAACCCCTATTTGTTTATTTTTCTAAATACATTC

REV round4-version1_poll_data

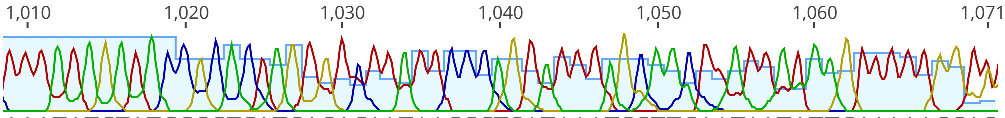

FWD HC00558165_4 AAATATGTATCCGCTCATGAGACAATAACCCTGATAAATGCTTCAATAATATTGAAAAAGGAG

REV round4-version1_poll_data
