# Supplementary material for: A digital DNA system favours the superiority of unidirectional inheritance over ‘Lamarckian’ inheritance
Source: PLoS Comput Biol. 2025 Oct 7;21(10):e1012677. doi: 10.1371/journal.pcbi.1012677 (PMC12517530; doi:10.1371/journal.pcbi.1012677)
Supplement: S4 File — (DOCX) [file pcbi.1012677.s009.docx]

**Supplementary File 4**

A digital DNA system reveals the superiority of unidirectional inheritance over ‘Lamarckian’ inheritance

**Aswathi Shiju^1,2^, Samantha D. M. Arras^2^, Allen G. Rodrigo^2^, Anthony M. Poole^1,2*^**

^1^Digital Life Institute & ^2^School of Biological Sciences, University of Auckland, Auckland, New Zealand.

*Correspondence: [a.poole@auckland.ac.nz](mailto:a.poole@auckland.ac.nz)

**Questionnaire:**

For each round, volunteers were sent .wav files for all five mutated versions of Ode to Joy (see main paper for details) and asked the following question:

*Which of the files (ROUND#_FILE1-5) did you like the best (or least dislike)?*

**Vote data – raw results:**

| Round 1 version # | Votes (N=12) |
| --- | --- |
| 1 | 7 |
| 2 | 2 |
| 3 | 1 |
| 4 | 0 |
| 5 | 2 |

| Round 2 version # | Votes (N=12) |
| --- | --- |
| 1 | 7 |
| 2 | 3 |
| 3 | 0 |
| 4 | 0 |
| 5 | 2 |

| Round 3 version # | Votes (N=12) |
| --- | --- |
| 1 | 5 |
| 2 | 3 |
| 3 | 0 |
| 4 | 0 |
| 5 | 4 |

| Round 4 version # | Votes (N=12) |
| --- | --- |
| 1 | 8 |
| 2 | 1 |
| 3 | 0 |
| 4 | 2 |
| 5 | 1 |
